# Association of Oral and General Health with Nutritional Status of Older Adults Attending Two Medical Centers in Riyadh, Saudi Arabia: A Cross-Sectional Study

**DOI:** 10.3390/nu15184032

**Published:** 2023-09-18

**Authors:** Alghaliyah A. Alghofaili, Alanoud I. Aladel, Abdullah M. Alsoghier, Fatmah Othman, Mustafa M. Shoqeair, Saud M. Alqahtani, Ali M. Alnughaimish, Badryh A. Alanazi, Sara A. AI Mosallam, Azzam S. Alharbi, Mohammed Alquraishi

**Affiliations:** 1 Community Health Sciences, College of Applied Medical Sciences, King Saud University, Riyadh 11451, Saudi Arabia or alghaliyah.alghofaili@gmail.com (A.A.A.); 439200179@student.ksu.edu.sa (B.A.A.); 439202863@student.ksu.edu.sa (S.A.A.M.); malquraishii@ksu.edu.sa (M.A.); 2 Department of Oral Medicine and Diagnostic Sciences, College of Dentistry, King Saud University, Riyadh 11451, Saudi Arabia; aalsoghier@ksu.edu.sa; 3 Epidemiology and Biostatistics Department, College of Public Health and Health Informatics, King Saud bin Abdulaziz University for Health Sciences (KSAU-HS), Riyadh 11426, Saudi Arabia; othmanf@ksau-hs.edu.sa; 4 King Abdullah International Medical Research Center, Riyadh 11481, Saudi Arabia; 5 Department of Clinical Nutrition, King Khalid University Hospital (KKUH), Riyadh 12372, Saudi Arabiaalqsaud@ksu.edu.sa (S.M.A.); anughaimish@ksu.edu.sa (A.M.A.); dt.azzam.18@gmail.com (A.S.A.)

**Keywords:** geriatric, malnutrition, nutritional status, oral health, quality of life

## Abstract

Malnutrition could profoundly affect older adults’ oral health and quality of life, whereas oral health might, in turn, impact dietary intake and nutritional status. The present study aimed to investigate the association between general and oral health and nutritional status among older adults attending nutrition clinics at two main medical centers in Riyadh, Saudi Arabia. A cross-section study was carried out among adult patients (≥60 years) who attended a geriatric clinic or nutrition clinic at King Khalid University Hospital or King Abdulaziz Medical City, Riyadh. A validated clinician’s Mini Nutritional Assessment Short-Form (MNA-SF), Oral Health Impact Profile-5 (OHIP-5), and 36-Item Short Form Survey (SF-36) were collected from each participant. A total of 261 participants with a mean age of 72.14 (±8.97) years were recruited. Diabetes (71%) and hypertension (80%) were present in the majority of patients. The overall MNA-SF score was (10 ± 3). Based on the categorization of the MNA-SF score, 65.9% were classified as malnourished or at risk of malnutrition. Participants with OHIP-5 scores higher than the median (>5) were more likely to be malnourished than those with scores at or lower than 5 (*p* < 0). The adjusted odd ratio for the MNA-SF score categories indicated that for a one-unit increase in the total SF-36 score, the odds of the malnourished category are 0.94 times less than the risk of malnutrition and normal nutritional status, with OR 0.97 (95% CI 0.94–0.95). Malnutrition or being at risk of malnutrition is likely associated with poor general and oral health. Healthcare providers need to incorporate dietitians into care plans to promote the nutritional health of older adults.

## 1. Introduction

A significant demographic trend that is occurring around the globe is the aging population. Older adults (defined as those 60 and older) [1] made up around 5.6% of the total population in the Kingdom of Saudi Arabia (KSA) in 2021 [2], and this proportion is anticipated to rise to 22.9% by 2050 [3]. Malnutrition, which is a nutritional condition with a negative functional or clinical impact on the body [4], is particularly common among older adults [5]. Moreover, malnutrition is linked to an increase in the risk of falls, loss of mobility, and poor wound healing in older adults [6] as well as higher healthcare expenses [7] and a poorer quality of life [8]. Several studies suggest that several factors may impact the nutritional status and good eating habits of older adults in the community [9,10,11]. Oral health, as one of the relevant aspects [11], is crucial to older adults’ nutritional status [12], with poor oral health often increasing the risk of being underweight [13]. The National Diet and Nutrition Survey (NDNS) [14] and the US National Health and Nutrition Examination Surveys (NHANES) [15,16] found a link between poor oral health and inadequate food intake in older adults. Poor dental health may impact food quality and nutrient intake, potentially increasing the risk of various systemic disorders [17]. A reduced number of teeth and posterior occluding teeth in this aging group are also demonstrated to impact chewing capacity, resulting in changed meal choices and reduced nutritional status [18]. Aside from clinical signs, the influence of quality-of-life dimensions, particularly general health and poor dental health, on nutritional status is significant in older adults.

At the national level, there are few studies that examine the impact of oral health on nutritional status, and some of those studies focus on hospitalized patients [19]. As the country is directed toward promoting the prevention of health risks, there is a need to address the relationship between oral health and the nutritional status of older adults. The aim of this study, therefore, was to assess the general and oral health and its relationship to nutritional status among older Saudi adults.

## 2. Materials and Methods

### 2.1. Study Design and Setting

A cross-sectional study was conducted among older adults attending the geriatric clinic or nutrition clinic at King Khalid University Hospital (KKUH) or King Abdulaziz Medical City (KAMC), Riyadh, KSA, between August 2022 and February 2023. KKUH is a governmental teaching hospital under the umbrella of the Ministry of Health and provides primary, secondary, and tertiary care to the Saudi population [20]. KAMC is a tertiary care facility with primary care clinics that provide healthcare services for eligible Saudi National Guard soldiers, employees, and their families [21]. 

### 2.2. Study Population

Saudi adult patients 60 years old or above attending geriatric or nutrition clinics at KKUH or KAMC were invited to participate in the study. Patients with a cancer diagnosis, with dysphagia resulting from stroke or musculoskeletal disease, receiving (full) enteral or parenteral tube feeding, and with cognitive impartment conditions were excluded from the study. A minimum sample of 232 participants was required to have an effect size of 0.25 [22], with alpha error and power probability of 0.05 and 0.90, respectively [22], and around 4% probability of multiple missing responses [23] using G*Power software (version 3.1.9.6) [24]. A non-random convenience sampling technique was used in recruiting the participants.

### 2.3. Study Tools

Standardized questionnaires consisting of three sections were personally administered to the participants or a guardian or caregiver who could answer the questions. The first one was the validated Mini Nutritional Assessment Short-Form (MNA-SF), which was used to assess the nutritional status. MNA-SF is a commonly used screening approach for detecting malnutrition in older adults and was previously used among the Saudi population [24,25]. It consists of six questions, a body mass index (BMI) evaluation, and a calf-circumference calculation if a BMI evaluation is impossible [26]. BMI was categorized as underweight (<18.5 kg/m^2^), normal weight (18.5–24.9 kg/m^2^), overweight (25.0–29.9 kg/m^2^), and obese (≥30.0 kg/m^2^).

Patients were classified as follows: malnourished category if the MNA-SF score ranged between 0 and 7, at risk of malnutrition category if the score ranged between 8 and 11, and normal nutritional status category if the MNA-SF score ranged between 12 and 14. The second part was the Arabic version of the health-related quality of life (HRQOL), which was used to assess the general-health status. This validated survey consists of a 36-Item Short Form Survey (SF-36). Each question in the form was scored, recorded, and averaged using scoring instructions provided by the RAND Corporation [27].

SF-36 scores range from 0 to 100; lower scores show more disability and higher scores show less disability. Many studies indicated good validity and reliability scores in its original English version as well as in the Arabic version [20,22].

Lastly, Oral Health Impact Profile-5 (OHIP-5) was used to evaluate the impact of oral illness on quality of life and measure the effects of clinical interventions. OHIP-5 has a minimum score of 5 and a maximum score of 25, with higher numbers indicating poorer oral health. Adding to the standardized survey, information on sociodemographic data, including age, gender, education, employment, marital status, and tobacco use, was collected. After completing the survey, the medical file of each patient was reviewed for clinical data, to extract data on chronic diseases (hypertension, diabetes, heart disease, presence of hypercholesterolemia, and any psychiatric illness) and a list of the current medication being received. 

### 2.4. Ethical Consideration

This study was approved by the Research Ethics Committee at KSU (ref.: 22/0086/IRB; study no.: E-22-6571) and the institutional review board (IRB) of King Abdullah International Medical Research Center (KAIMRC) (ref.: IRB/1433/22; study no.: NRC22R/300/07). Participation was voluntary, and patients completed informed consent before completing the survey. 

### 2.5. Statistical Analysis

The collected data were entered into MS Excel. Descriptive statistics were used to describe the demographic and clinical characteristics of the study population. Continuous variables were described as mean and standard deviation (SD) if normally distributed, otherwise median and interquartile ranges were used. Shapiro–Wilk test was performed to check the normality of the data, and *p* > 0.05 was considered as evidence of non-normality. For categorical variables, frequencies and percentages were used. The association between participants’ sociodemographic characteristics and oral health responses with nutritional status categories was assessed using chi-square (or Fisher’s exact test) and ANOVA, as appropriate. The oral health variable was categorized into two categories based on the distribution of the OHIP-5 score. The score of general health was dealt with as a continuous variable. Ordinal logistic regression was used to assess the association between the three MNA-SF score categories and general health and was presented as odds ratio (OR) and 95% confidence interval (CI) using “ologit command” followed by “or” option. Similarly, logistic regression was used to assess the association between two OHIP-5 categories and the general-health score. 

The interpretation needs to consider that lower HRQOL scores indicate more disability and higher scores indicate less disability, while OHIP-5 has a minimum score of 5 and a maximum score of 25, with higher numbers indicating poorer oral health. A *p* of less than 0.05 was set as a cut-off for statistical significance. All analyses were completed with Stata 15 software system (Stata Corp L.P., College Station, TX, USA).

## 3. Results

### 3.1. Description of the Study Population and Its Nutritional Status

A total of 261 participants were included in the analysis. The mean age of study participants, of which 50% were males, was 72.1 ± 8.9 years. Table 1 provides the demographic characteristics of the study population. The majority were married (60.9%), were living in the central region (82%), and had an elementary school degree or lower (65.2%). And 93% of the participants had at least one chronic disease, of which 80% had hypertension, 71.3% had type 2 diabetes, and 95% were on long-term medication. The overall MNA-SF score was 10 ± 3. Based on the categorization of the MNA-SF score, 65.9% were classified as malnourished or at risk of malnutrition, with the remaining 34.1% (*n* = 89) classified as having normal nutritional status. In addition, 50% (*n* = 139) of the study population had a moderate-to-severe decrease in food intake, whereas 42% (*n* = 110) had a weight loss of more than 3 kg in the last 3 months. 

Table S1 shows the nutritional status of the participants according to the MNA-SF questionnaire. The nutritional status varied with the different age groups, in which 16% of participants aged above 75 years were at risk of malnutrition, and 11% of them were malnourished (Table 1). The percentage of female participants at risk of malnutrition group was higher compared to the percentage of male participants (57% vs. 43%; *p* = 0.041). The educational level and marital status were different across the three nutritional status groups (Table 1). Based on the BMI and MNA-SF classification, over 90% (*n* = 46) of malnourished people were categorized as having a BMI above 18.5 kg/m^2^. 

Regarding comorbidities, 84% of participants in the malnourished category had type 2 DM compared to 69% and 67% of participants in the normal nutritional and at risk of malnutrition groups, respectively, with *p* = 0.059. For psychiatric illness, 23% of participants in the malnourished category had psychiatric illness compared to 3% and 9% in the normal nutritional and at risk of malnutrition groups, respectively, with *p* < 0.001.

### 3.2. Oral Health Status of Participants and Its Association with Nutritional Status

Based on the responses to each item on the OHIP-5, 23.4% of participants reported having difficulties chewing often/very often. The response to each question on the OHIP-5 scale is provided in Table S2. The overall OHIP-5’s total mean score was 8.77 ± 5.12. Based on the distribution of the total OHIP-5 score, the Shapiro–Wilk test for normal data was statistically significant (*p* value < 0.001), indicating that it is not normally distributed with a median of 5 (IQR 5-11). Based on this, the response of the study population was recategorized into two categories (below or equal to the median and above the median). Around 136 (52.1%) participants in the study population had an OHIP-5 score of 5 or below, whereas 125 (47.8%) scored above 5 in the OHIP-5 score category. 

The association between the OHIP-5 score categories and the three MNA-SF scores is presented in Table 2. Participants who had OHIP-5 scores above the median were more likely to be malnourished compared to participants who had OHIP-5 scores below or equal to 5, with *p* <0.001. The unadjusted OR for the MNA-SF score categories indicated that patients who had OHIP-5 scores above the median were three times more at risk of malnutrition compared to those who had OHIP-5 scores below or equal to the median (OR 3.07; 95% CI (1.90–4.96)), and this remained significant after adjusting for the confounder (Table 2).

### 3.3. Association between General Health and Nutritional Status

The median score of the HRQOL domains was 34 (IQR 23-50). Table 2 shows the median scores for the SF-36’s eight scales according to the MNA-SF score categories. The median of the SF-36 total scores across the three MNA-SF scores groups was significantly different (*p* < 0.001). Table S3 shows all the SF-36 questions for the general-health domain and the normal nutritional, at risk of malnourishment, and malnourished groups’ respective mean scores. The unadjusted OR for MNA-SF score categories indicated that for a one-unit increase in the total HRQOL score, the odds of the malnourished category are 0.94 times less than those of the risk of malnutrition and normal nutritional status categories, with OR 0.97 (95% CI 0.96–0.97) (Table 2). This remains significant even after adjusting the model for age, gender, marital status, and education status.

### 3.4. Relationships between Oral Health and General Health

The association between the OHIP-5 categories and the SF-36 domains is presented in Table 3. Participants who had a one-unit increase in the total HRQOL score are 0.97 times less likely to be in the above OHIP-5 median score category, with OR 0.97 (95% CI 0.96–0.98) (Table 2). This remains significant even after adjusting the model for age, gender, marital status, and education status.

## 4. Discussion

### 4.1. Main Findings

The present study indicated that oral health and general health were risk factors for nutritional status in older adults receiving clinical nutrition care. Moreover, the patients’ age, gender, health status (T2DM, psychiatric illness), educational level, and marital status were significantly associated with their nutritional status.

Previous studies indicated that malnutrition affects up to 75% of older adults globally [28]. The incidence of malnourished patients reached a high documented level in our study, two-thirds of older adults, as reported in a multilateral investigation [29]. Furthermore, another study reported a comparable prevalence rate of malnutrition (29%) and risk of malnutrition (47.6%) among aged Saudi Arabian patients, which is remarkably close to the findings of our study [24]. In contrast, a study conducted at King Abdul Aziz University Hospital’s geriatric outpatient clinic in Jeddah stated that only 5.3% of the population was malnourished compared to 32.9% had an increased malnutrition risk [30]. This might be related to different research methods, such as the smaller sample size, which was avoided in the present study. According to these findings, malnutrition is quite common among older adults. This high prevalence might be ascribed to age-related comorbid medical issues, which can lead to inadequate food intake. Previous studies also reported similar results regarding the prevalence of malnutrition among overweight and obese older adults. In our study, we discovered that older adults with a BMI higher than 25 made up around 47% of malnourished people, which is consistent with a prior study that found 49% of older adults were malnourished and had a BMI higher than 25 [31].

OHRQOL is another factor that leads to geriatric malnutrition. It was discovered to be highly related to the risk of malnutrition in the present study. A systematic review indicated that malnutrition is related to the state of the hard and soft tissues of the mouth, the salivary flow, and xerostomia [32]. Limited evidence is available on the relationship between OHRQOL and malnutrition in older adults. A cross-sectional study conducted in Malaysia aimed to determine the association between OHRQOL and nutritional status in older adults and found that it was significantly associated with the nutritional condition of the respondents (OR = 2.3; *p* < 0.01) [33]. Another study conducted in Germany aimed to assess oral health, nutritional condition, and OHRQOL [34] found that nutritional status was influenced by missing teeth (β: −11.9; 95% CI: −6.4–−1.9; *p* < 0.01) and OHRQOL (β: −0.2; 95% CI: −0.1–0.0; *p* = 0.05).

Due to its implications regarding health and nutritional status, health-related quality of life (HRQOL) has attracted increased attention [35,36]. Health professionals have increasingly used HRQOL instruments over the past decade in assessing health status and outcomes. The SF-36 questionnaire [37], a widely used instrument for assessing general-health status, is used to assess HRQOL. The SF-36 scoring system consists of 36 questions divided into eight domains: physical functioning, limitations due to physical health, limitations due to emotional problems, energy/fatigue, emotional well-being, social functioning, pain, and general health. Several studies using different instruments found a link between the risk of malnutrition and a lower HRQOL and health status (*p* = 0.043, <0.001, respectively) [38,39]. A cross-sectional study assessing the relationship between nutritional status and HRQOL using the same present instruments (MNA-SF and SF-36) demonstrated that malnutrition is associated with a lower HRQOL in older adults [40], which aligns with the current findings. This is also supported by the results of this study, which sought to investigate the correlations between nutritional status and HRQOL (*p* < 0.05) [41]. On the other hand, another study indicated no direct relationship between malnutrition and HRQOL in older adults using the EuroQol five dimensions (EQ-5D) questionnaire for measuring HRQOL and BMI with specific parameters (hand-grip strength, self-reported appetite, and swallowing problems) for measuring nutritional status [42].

### 4.2. Strengths and Limitations

The present study was the first to assess the general- and oral-health-related quality of life and nutritional status among older Saudi adults. It included a relatively large sample size and used validated patient- and clinician-based instruments like OHIP-5 and MNA-SF. The cross-sectional nature of this study was the main limitation, since it was impossible to determine whether the exposure or result occurred first. Therefore, interventional studies with longitudinal follow-up are needed to indicate a causal relationship between common oral health issues (e.g., dental caries, periodontitis, and teeth loss) and nutritional status.

Furthermore, the heterogeneity of the research participants, with diverse medical problems at different clinical care stages, could limit the present findings’ generalizability. Moreover, “malnutrition/risk” was determined using MNA-SF as a screening tool, rather than evaluating food intake in detail and assessing nutritional markers in the blood. Nonetheless, the MNA-SF remains one of the most widely used measures for evaluating nutrition in older adults [43]. In addition, there is no single instrument available to accurately determine nutritional status [44].

### 4.3. Implications for Practice and Future Direction

The malnourished older adults had significantly poorer oral health and HRQOL. As a result, older adults, particularly those with prosthodontic problems, should receive nutritional assessments, an intervention, and graded management as soon as possible to improve their nutritional and health status [45]. Consequently, oral and general health clinicians in the care of patients with malnutrition may consider a multidisciplinary approach toward nutritional-status promotion by incorporating nutritionists into the patient’s care plans.

## 5. Conclusions

A lack of evidence that evaluated the link between general and oral health and nutritional status in older adults using the same methods was found in the literature review. The current study’s findings indicated that malnutrition and the risk of malnutrition were prevalent among older adults and could be predicted using self-reported oral- and general-health-related quality of life status. Future research could focus on prospectively designed interventions to promote nutritional status through oral health promotion and interventions (e.g., dental and periodontal rehabilitation).

## Figures and Tables

**Table 1:** The participants’ sociodemographic characteristics and oral health status were based on the MNA-SF scores.

			Nutritional Status Based on MNA-SF Scores			
Study Variables		Total Number (%)	Normal Nutritional Status N (%)	At the Risk of Malnutrition N (%)	Malnourished N (%)	
Age category *						0.054
	60–64	61 (23.4)	25 (9.6)	28 (10.7)	8 (3.1)	
	65–69	59 (22.6)	24 (9.2)	27 (10.3)	8 (3.1)	
	70–74	44 (16.9)	16 (6.1)	22 (8.4)	6 (2.3)	
	>75	97 (37.2)	24 (9.2)	44 (16.9)	29 (11.1)	
Gender *	Male	132 (50.6)	54 (60.7)	52 (43)	26 (51)	0.041
	Female	129 (49.4)	35 (39.3)	69 (57)	25 (49)	
BMI category *	Underweight	6 (2.3)	0 (0)	1 (0.8)	5 (9.8)	0.003
	Normal	77 (29.5)	23 (25.8)	32 (26.4)	22 (43.1)	
	Overweight	70 (26.8)	26 (29.2)	35 (29)	9 (17.7)	
	Obese	108 (41.3)	40 (45)	53 (43.8)	15 (29.4)	
Weight (kg) (mean ± SD)		74.1 ± 16.9	76.8 ± 14.7	75.6 ± 16.8	65.9 ± 18.4	<0.001
Height (cm) (mean ± SD)		159 ± 9.2	160.4 ± 9.17	160.1 ± 9.5	158 ± 28.3	0.358
BMI (kg/m^2^) (mean ± SD)		29.1 ± 7.0	30.1 ± 6.5	29.6 ± 6.9	26.4 ± 7.49	0.007
Comorbidity	Hypertension	209 (80.1)	67 (75.3)	98 (81)	44 (86.3)	0.305
	Heart diseases	80 (30.65)	24 (27)	35 (29)	21 (41.2)	0.197
	Hypercholesterolemia	158 (60.5)	48 (54)	78 (64.5)	32 (62.7)	0.298
	T2DM *	186 (71.3)	62 (69.6)	81 (67.0)	43 (84.3)	0.059
	Psychiatric illness *	26 (10)	3 (3.4)	11 (9)	12 (23.5)	0.001
Taking long-term medications	Yes	248 (95)	86 (96.6)	113 (93.4)	49 (96)	0.525
	No	13(5)	3(23)	8(61.5)	2(15.3)	
Marital status *	Married	159 (60.9)	63 (24.1)	65 (24.9)	31 (11.9)	0.042
	Other	102 (39.1)	26 (10.0)	56 (21.5)	19 (7.3)	
Educational level *	Illiterate	85 (32.6)	18 (6.9)	42 (16.1)	25 (9.6)	0.011
	Elementary school	85 (32.6)	29 (11.1)	40 (15.3)	16 (6.1)	
	Middle/high school	57 (21.8)	25 (9.6)	26 (10.0)	6 (2.3)	
	Bachelor’s and above	34 (13)	17 (6.5)	13 (5.0)	4 (1.5)	
Employment status	Still working	10 (3.9)	5 (1.9)	5 (1.9)	0 (0.0)	0.280
	Retired/unemployed	251 (69.1)	84 (32.2)	116 (44.4)	51 (19.5)	
Smoking	Non-smoker	205 (78.5)	70 (26.8)	95 (36.4)	40 (15.3)	0.937
	Ex-smoker	34 (13)	11 (4.2)	15 (5.7)	8 (3.1)	
	Smoker	22 (8.4)	8 (3.1)	11 (4.2)	3 (1.1)	

(MNA-SF): Mini Nutritional Assessment Short-Form. The patients were classified as malnourished if they received a score between 0 and 7 on the MNA-SF scale. The patients were classified as at risk of malnutrition if they received a score between 8 and 11 on the MNA-SF scale. The patients were classified as having normal nutritional status if they received a score between 12 and 14 on the MNA-SF scale. * A *p*-value of <0.05 was considered significant. BMI was categorized as underweight (<18.5 kg/m^2^), normal weight (18.5–24.9 kg/m^2^), overweight (25.0–29.9 kg/m^2^), and obese (≥30.0 kg/m^2^).

**Table 2:** Relationships between OHIP-5 and SF-36 scores for each domain and nutritional status based on MNA-SF scores of the participants.

	MNA-SF Score Categories			OR (95% CI)	Adjusted OR * (95% CI)
	Normal Nutritional Status	At Risk of Malnutrition	Malnourished		
OHIP-5 ^1^ categories					
≤to median	136 (52.1)	60 (67.4)	63 (52.1)	Reference	Reference
>than median	125 (47.8)	29 (32.5)	58 (47.9)	3.07 (1.90–4.96)	2.57 (1.53–4.31)
36-Item Short Form Survey domains ^2^					
Physical functioning (median IQR)	50 (25–75)	25 (10–60)	0 (0–20)	0.97 (0.96–0.97)	0.97 (0.96–0.98)
Limitations due to physical health (mean ± SD)	35.6 (21)	15.9 (18)	4.4 (4.3)	0.98 (0.97–0.99)	0.98 (0.97–0.99)
Limitations due to emotional problem (mean ± SD)	63 (15)	51 (17)	44 (18)	0.98 (0.97–0.99)	0.98 (0.97–0.99)
Energy/fatigue (median IQR)	45 (40–60)	35 (25–45)	25 (10–35)	0.93 (0.92–0.95)	0.94 (0.92–0.95)
Emotional well-being (median IQR)	60 (52–76)	48 (40–60)	44 (32–56)	0.95 (0.94–0.97)	0.95 (0.94–0.97)
Social functioning (median IQR)	75 (50–100)	50 (37.5–75)	25 (0–37)	0.96 (0.95–0.97)	0.96 (0.95–0.97)
Pain (median IQR)	57.5 (45–70)	45 (25–57.5)	32 (10–45)	0.96 (0.95–0.97)	0.96 (0.95–0.97)
General health (median IQR)	50 (45–55)	45 (40–50)	40 (30–45)	0.93 (0.91–0.95)	0.94 (0.92–0.96)
Total (median IQR)	47(36–69)	31 (24–44)	22 (13–26)	0.94 (0.92–0.955)	0.94 (0.93–0.95)

^1^ OHIP-5: Oral Health Impact Profile-5. The oral health variable were categorized into two categories based on the median score: below or equal to the median and above the median. ^2^ SF-36: 36-Item Short Form Survey. Scores range from 0 to 100; lower scores show more disability, and higher scores show less disability. * Adjusted for age, gender, marital status, and education level. The Shapiro–Wilk test was performed to check the normality of the distribution of the parameters and was based on the *p*-value of the test; the mean and SD were used for normal distribution, while median and IQR were used otherwise. SD: standard deviation; IQR: interquartile range; OR: odds ratio; CI: confidence interval.

**Table 3:** Relationships between SF-36 scores for each domain and OHIP-5 categories.

	OHIP-5 Categories			
36-Item Short Form Survey Domains ^1^	≤Median Score	>Median Score	OR (95% CI)	Adjusted OR * (95% CI)
Physical functioning (median IQR)	47.5 (20–70)	10 (0–40)	0.97 (0.96–0.98)	0.98 (0.97–0.99)
Limitations due to physical health (mean ± SD)	25.1 (11)	15.2 (19)	0.99 (0.98–1.00)	0.99 (0.98–1.11)
Limitations due to emotional problem (mean ± SD)	27 (19)	31 (17)	1.01 (0.99–0.1.10)	1.00 (0.99–1.9)
Energy/fatigue (median IQR)	40 (30–55)	30 (15–45)	0.97 (0.95–0.98)	0.97 (0.96–0.99)
Emotional well-being (median IQR)	56 (44–68)	52 (40–64)	0.98 (0.97–0.99)	0.98 (0.96–0.99)
Social functioning (median IQR)	62.5 (50–93.7)	37.5 (25–62)	0.97 (0.96–0.98)	0.97 (0.96–0.98)
Pain (median IQR)	47.5 (45–67.5)	32.5 (22.5–45)	0.97 (0.96–0.98)	0.97 (0.96–0.98)
General health (median IQR)	45 (45–50)	45 (35–50)	0.96 (0.94–0.98)	0.97 (0.94–0.99)
Total (median IQR)	39 (29–57)	27 (20–41)	0.97 (0.95–0.98)	0.97 (0.96–0.99)

^1^ SF-36: 36-Item Short Form Survey. Scores range from 0 to 100; lower scores show more disability, and higher scores show less disability. * Adjusted for age, gender, marital status, and education level. OHIP-5: Oral Health Impact Profile-5. The oral health variable was categorized into two categories based on the median score into two categories: below or equal to the median and above the median. The Shapiro–Wilk test was performed to check the normality of the distribution of the parameters and based on the *p*-value of the test, the mean and SD were used for normal distribution, while median and IQR were used otherwise. SD: standard deviation; IQR: interquartile range; OR: odds ratio; CI: confidence interval.

## Data Availability

Data are available upon request from A.I.A. (e-mail: aaladel@ksu.edu.sa).

## Supplementary Materials

The following supporting information can be downloaded at https://www.mdpi.com/article/10.3390/nu15184032/s1. Table S1: Mini Nutritional Assessment Short-Form results for each question; Table S2: OHIP-5 scale scores and nutritional status of the subjects; Table S3: All the 36-SF questions for the general health domain and for the normal nutritional, at risk of malnourishment, and malnourished groups’ respective mean scores.
