# Supplementary material for: Association of Oral and General Health with Nutritional Status of Older Adults Attending Two Medical Centers in Riyadh, Saudi Arabia: A Cross-Sectional Study
Source: Nutrients. 2023 Sep 18;15(18):4032. doi: 10.3390/nu15184032 (PMC10534773; doi:10.3390/nu15184032)
Supplement: Supplementary file 1 [file nutrients-15-04032-s001.zip › nutrients-2547109-supplementary.pdf]

Table S1: Mini Nutritional Assessment results for each question

Item of the questionnaire	Number (%)	
**Has food intake declined over the past 3 months due to loss of appetite, digestive problems, chewing or swallowing difficulties?**		
Severe decrease in food intake	63(24.1)	
Moderate decrease in food intake	76(29.1)	
No decrease in food intake	122(46.8)	
**Weight loss during the last 3 months**		
Weight loss greater than 3 kg (6.6 lbs)	110(42.1)	
Does not know	21(8.0)	
Weight loss between 1 and 3 kg (2.2 and 6.6 lbs)	24(9.2)	
No weight loss	106(40.6)	
**Mobility**		
Bed or chair bound	69(26.4)	
Able to get out of bed / chair but does not go out	49(18.8)	
Goes out	143(54.8)	
**Has suffered psychological stress or acute disease in the past 3 months?**		
Yes	54(20.7)	
No	207(79.3)	
**Neuropsychological problems**		
Severe dementia or depression	19(7.3)	
Mild dementia	34(13)	
No psychological problems	208(79.7)	
**Body Mass Index (BMI) (weight in kg) / (height in m)^2**		(mean ±SD)
BMI less than 19	7(2.7)	16.9±1.9
BMI 19 to less than 21	13(5.1)	20.2±0.6
BMI 21 to less than 23	24(9.2)	21.8±0.5
BMI 23 or greater	217(83.1)	30.9±6.3
**Overall Total (mean +/-SD)**	(10 ± 3)	
**MNA Category**		(mean ±SD)
**Normal nutritional status**	89(34.1)	13.2±0.8
**At risk of malnutrition**	121(46.4)	9.5±1.0
**Malnourished**	51(19.5)	5.4±1.6

Table S2: OHIP-5 scale scores and nutritional status of the subjects*

the Oral Health Impact Profile	Total number (%)	MNA score			P value
		Normal nutritional status **Number (%)**	At risk of malnutrition **Number (%)**	Malnourished **Number (%)**	
Have you had difficulty chewing any foods because of problems with your teeth, mouth, dentures, or jaw?					
Total OHIP-5 score					
Never	142(54.4)	63(70.7)	64(52.80	15(29.4)	<0.001
Hardly ever	11(4.2)	3(3.3)	6(4.9)	2(3.9)	
Occasionally	47(18)	14(15.7)	24(19.8)	9(17.6)	
Often	30(11.5)	7(7.8)	12(9.9)	11(21.5)	
Very often	31(11.9)	2(2.2)	15(12.4)	14(27.4)	
Have you had painful aching in your mouth?					
Never	162(62)	67(75.2)	73(60.3)	22(43.1)	<0.001
Hardly ever	13(5)	4(4.4)	7(5.7)	2(3.9)	
Occasionally	48(18.4)	12(13.4)	23(19.0)	13(25.4)	
Often	23(8.8)	6(6.7)	7(5.7)	10(19.6)	
Very often	15(5.8)	0(0)	11(9.0)	4(7.8)	
Total Number (Mean±SD)		89(1.5±0.9)	121(1.9±1.4)	51(2.4±1.4)	
Have you felt uncomfortable about the appearance of your teeth, mouth, dentures, or jaws?					
Never	190(72.8)	73(82.0)	86(71.0)	31(60.7)	0.008
Hardly ever	20(7.7)	7(7.8)	9(7.4)	4(7.8)	
Occasionally	20(7.7)	4(4.4)	9(7.4)	7(13.7)	
Often	15(5.7)	4(4.4)	6(4.9)	5(9.8)	
Very often	16(6.1)	1(1.1)	11(9.0)	4(7.8)	
Total Number (Mean±SD)		89(1.3±0.85)	121(1.7±1.3)	51(2±1.4)	
Have you felt that there has been less flavour in your food because of problems with your teeth, mouth, dentures, or jaws?					
Never	200(76.6)	78(87.6)	93(76.8)	29(56.8)	<0.001
Hardly ever	15(5.7)	5(5.6)	4(3.3)	6(11.7)	
Occasionally	20(7.7)	2(2.2)	11(9.0)	7(13.7)	
Often	15(5.7)	4(4.4)	6(4.9)	5(9.8)	
Very often	11(4.2)	0(0)	7(5.7)	4(7.8)	
Total Number (Mean±SD)		89(1.2±0.7)	121(1.6±1.3)	51(2±1.3)	
Have you had difficulty doing your usual jobs because of problems with your teeth, mouth, dentures, or jaws?					
Never	210(80.5)	78(87.6)	98(80.9)	34(66.6)	<0.001
Hardly ever	17(6.5)	6(6.7)	11(9.0)	0(0)	
Occasionally	13(5)	2(2.2)	3(2.4)	8(15.6)	
Often	12(4.6)	3(3.3)	5(4.1)	4(7.8)	
Very often	9(3.4)	0(0)	4(3.3)	5(9.8)	

Table S3 shows all the 36-SF questions for the general health domain and for the normal, at-risk, and malnourished groups' respective mean scores

36 Health Survey Questionnaire		Normal nutritional status (Mean±SD)	At risk of malnutrition (Mean±SD)	Malnourished (Mean±SD)	p value
**Physical Health**					
**Physical Functioning (PF)**					
3a	Vigorous activities	21.9±31.9	13.6±26.6	1.9±9.8	<0.001
3b	Moderate activities	43.8±40.4	24.3±34.2	5.88±19.0	<0.001
3c	Lift, Carry Groceries	59.5±39.0	43.3±40.7	14.7±28.7	<0.001
3d	Climb Several flights	40.4±40.5	20.2±33.8	5.88±16.2	<0.001
3e	Climb One flight	58.9±42.3	33.8±40.9	12.7±29.7	<0.001
3f	Bend, Kneel	59.5±37.5	40.9±36.5	21.5±32.0	<0.001
3g	Walk mile	31.4±40.1	19.0±33.0	5.88±19.0	<0.001
3h	Walk Several blocks	43.8±41.8	26.0±38.2	8.8±21.6	<0.001
3i	Walk one Block	68.5±37.2	45.8±41.6	15.6±29.1	<0.001
3j	Bathe, Dress	82.5±30.2	61.5±39.1	27.4±37.8	<0.001
**Role-Physical (RP)**					
4a	Cut Down time	38.2±48.8	16.5±37.2	3.9±19.6	<0.001
4b	Accomplished Less	34.8±47.9	14.8±35.7	3.9±19.6	<0.001
4c	Limited in kind	35.9±48.2	16.5±37.2	5.8±23.7	<0.001
4d	Had difficulty	33.7±47.5	15.7±36.5	3.9±19.6	<0.001
**Bodily Pain (BP)**					
7	Pain-Magnitude	46.9±25.2	33.5±22.6	28.6±23.0	<0.001
8	Pain-interfere	71.9±24.3	50.6±31.5	28.4±23.4	<0.001
**General Health (GH)**					
1	EVGFP rating	47.7±22.8	32.0±24.6	23.0±21.1	<0.001
11a	Sick Easier	51.1±17.6	48.5±21.4	35.7±23.5	<0.001
11b	As healthy	50.5±19.2	44.8±20.1	44.6±22.5	0.0939
11c	Health to get worse	53.0±16.7	52.2±15.8	44.6±24.1	0.0176
11d	Health Excellent	55.8±16.4	49.5±22.5	38.2±22.5	<0.001
**Mental Health**					
**Vitality (VT)**					
9a	Pep/life	38.2±27.2	24.4±24.0	9.8±12.8	<0.001
9e	Energy	35.0±28.3	20.6±26.3	17.2±26.2	<0.001
9g	Worn out	67.8±25.1	51.0±26.5	35.2±28.1	<0.001
9i	Tired	59.1±21.7	43.8±21.9	29.8±25.4	<0.001
**Social Functioning (SF)**					
6	Social-Extent	74.1±23.3	53.9±31.1	26.4±26.6	<0.001
10	Social-Time	71.6±24.7	54.5±30.7	25.9±29.1	<0.001
**Role-Emotional (RE)**					
5a	Cut down time	44.9±50.0	26.4±44.2	9.8±30.0	<0.001
5b	Accomplished less	46.0±50.1	27.2±44.7	11.7±32.5	<0.001
5c	Not careful	46.0±50.1	23.9±42.8	11.7±32.5	<0.001
**Mental Health (MH)**					
9b	Nervous	67.6±24.7	60.1±26.6	54.5±33.4	0.0194
9c	Down in dumps	81.7±21.8	63.6±28.1	46.6±30.6	<0.001
9d	Peaceful	45.8±27.0	40.6±25.0	45.4±28.0	0.3028
9f	Blue/Sad	74.8±21.2	59.8±28.4	45.0±30.4	<0.001
9h	Happy	45.6±27.3	32.8±26.4	28.2±23.0	<0.001
